# Use of Blood Powder (Ground and Irradiated) for the Manufacture of Chocolate Agar

**DOI:** 10.3390/ijms24097965

**Published:** 2023-04-27

**Authors:** Pablo Casino, Asunción López, Sara Peiró, Inés Terrones, Gemma Agustí, Daniela Terlevich, Daniel Asensio, Ana María Marqués, Núria Piqué

**Affiliations:** 1 Department of Quality Control, Reactivos Para Diagnóstico, S.L. (RPD), Josep Tura, 9H, Polígon Industrial Mas D’en Cisa, Sentmenat, 08181 Barcelona, Catalonia, Spain; pacasinoalcalde45@gmail.com (P.C.);; 2 Microbiology Section, Department of Biology, Healthcare and Environment, Faculty of Pharmacy and Food Sciences, Universitat De Barcelona (UB), 08028 Barcelona, Catalonia, Spain; 3 Institut De Recerca En Nutrició I Seguretat Alimentària De La UB (INSA-UB), Universitat De Barcelona, 08921 Barcelona, Catalonia, Spain

**Keywords:** chocolate agar, defibrinated blood, hemoglobin, powder blood, hemin, autoclaved, gamma irradiation, grinding

## Abstract

Chocolate agar (CA) is an enriched medium for the isolation and identification of fastidious bacteria. Defibrinated blood is used to manufacture CA, but this expensive product is not always affordable for companies in developing countries. Blood powder (BP) is potentially a cheaper alternative, although its pre-treatment using autoclaving can impair the quality of the media. Therefore, optimization of BP as a substitute for defibrinated blood for CA manufacture deserves further research. CA was manufactured with irradiated BP (dehydrated bovine blood powder) and its physical and microbiological characteristics were compared with those of conventional CA and CA prepared with autoclaved BP. Each medium was seeded with 20–200 CFU of target bacteria using the spiral pouring method. Finally, another medium was prepared using BP pre-treated by grinding and gamma irradiation and its performance assessed. Compared to conventional CA, the medium containing ground and irradiated BP provided a similar CFU count for both fastidious (*Neisseria*, *Haemophilus*, *Campylobacter*, and *Streptococcus*) and non-fastidious (*Moraxella*, *Staphylococcus*, *Enterococcus*, *Klebsiella*, and *Pseudomonas*) species, unlike the medium prepared with BP subjected only to irradiation, which provided a lower growth of fastidious species. Morphology and characteristics of all bacterial colonies were very similar in conventional CA and the new medium, the number of *Pseudomonas* CFU being higher in the latter. The medium prepared with ground plus irradiated vs. irradiated BP more closely resembled conventional CA, having a browner background. The new CA medium prepared with ground and gamma irradiation-sterilized BP has comparable productivity properties to conventional CA. Therefore, it could be a more practical and economical methodology to facilitate large-scale CA manufacture.

## 1. Introduction

Blood is used as an essential nutritional supplement in microbiological culture media, particularly to culture and isolate fastidious microorganisms such as *Streptococcus* spp., *Haemophilus* spp., or *Neisseria* spp. for the diagnosis of serious bacterial infections [1,2]. When blood cultures are flagged as positive, they are seeded on solid media to produce enough bacterial biomass for identification and susceptibility testing [3]. The most frequently used solid media containing blood are blood agar and chocolate agar (CA). Blood agar is prepared by the addition of sterile defibrinated blood after the mixture of nutrients and agar is autoclaved and cooled to 45–60 °C. Then, the medium is immediately dispensed onto plates [4]. Chocolate agar is prepared by adding defibrinated blood to the warm base media (>60 °C) and gently shaking the mixture for 15–20 min, promoting the hemolysis of erythrocytes and the release of hemin (X factor) and nicotinamide adenine dinucleotide (NAD) [4,5], which are particularly required for the growth of *Haemophilus* spp. and *Neisseria* spp. [6]. The name derives from the chocolate-brown color acquired by the medium after red blood cell (RBC) lysis.

Sheep, horse, and goat blood are the most used for both types of media, although cattle and pig blood are possible alternatives [1,4,6,7]. In developing countries, human blood is still used to manufacture blood agar media, despite data suggesting it produces poor bacterial isolation and may inhibit growth due to antibody content [4,8,9,10]. The economic and handling advantages of using expired human blood donated for transfusions favors its use in countries in the Asia–Pacific Region, where high temperatures and conservation costs hinder blood handling [8,11].

The cost of producing blood-based media is increased by defibrination, in which a portion of the plasma component is separated from the RBCs to form a purified RBC suspension. Defibrination through agitation is currently regarded as the best method to prevent blood clotting, as other options, including the use of anticoagulants such as citric acid, can inhibit bacterial growth [5,12,13]. The specialized equipment required to produce defibrinated blood and transportation costs limit the viability of this option in developing countries [7,11,14].

Given that the essential component of chocolate agar for growth efficiency is hemoglobin (Hb) [15], dehydrated blood powder (BP) has been used as an alternative ingredient, being much less perishable and easier to handle than fresh defibrinated blood [4]. Before adding this product to the medium mix, it is necessary to dissolve the powder and sterilize it using autoclaving [4] to promote the release of the growth factors. However, the autoclaving conditions negatively affect the physical properties of BP and the final media, resulting in the formation of precipitates and lack of homogeneity.

To overcome these drawbacks, considering that evidence indicates that BP is a suitable substitute for fresh defibrinated blood for CA manufacture, we hypothesized that BP could be treated using gamma irradiation instead of autoclaving. In this study, after preparing a new CA medium with ground and gamma irradiation-sterilized BP, we assessed if the physical and productivity properties of the medium were appropriate for large-scale production and could, thus, be a substitute for the use of perishable defibrinated blood.

## 2. Results

### 2.1. Comparison of Chocolate Agar Prepared with Autoclaved or Irradiated BP

CA manufactured with BP sterilized by autoclaving (CA-PT) or using irradiation (CA-PR) was compared with conventional CA to assess the productivity properties of the media. Visually, the main difference between CA-PT and CA-PR media was the appearance of small precipitates in CA-PT. Both CA-PT and CA-PR had a more reddish background than CA.

**Figure 1:**
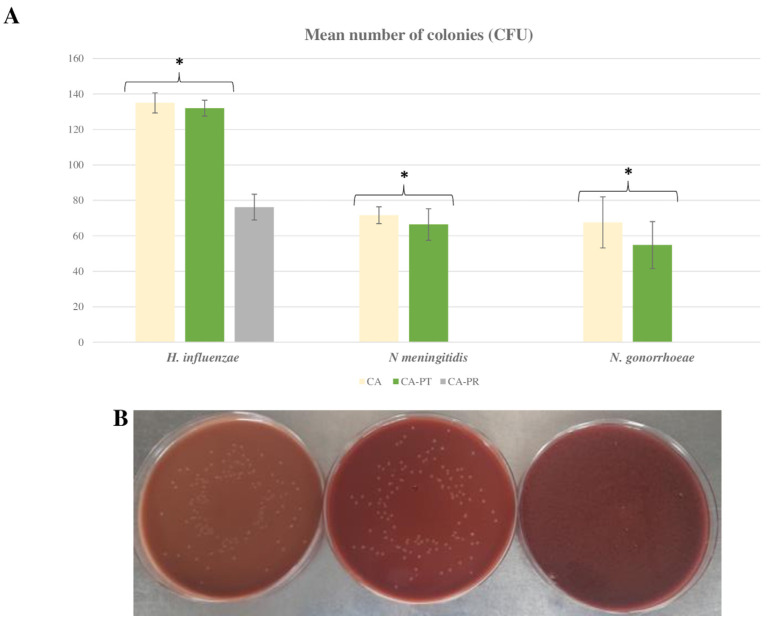
Growth of *Haemophilus influenzae* (ATCC 10211), *Neisseria* (*N. meningitidis* ATCC 13090), and *N. gonorrhoeae* ATCC 19424) species on CA, CA-PT, and CA-PR media. (**A**) Bar graph showing the colony count (CFU). (**B**) *Haemophilus influenzae* growing on chocolate agar media (CA-left, CA-PT-center, and CA-PR-right). * *p* < 0.05 (CA and CA-PT vs. CA-PR).

Regarding microbial growth, statistically significant lower CFU counts were obtained in CA-PR than in the other culture media for all tested bacteria (*Haemophilus* spp. and *Neisseria* spp.) (*p* < 0.01) (Figure 1). When comparing CA and CA-PT, no significant differences were observed in the mean CFU of *Haemophilus influenzae* (*p* = 0.061), *Neisseria meningitidis* (*p* = 0.28), and *Neisseria gonorrhoeae* (*p* = 0.18). Regarding colony size, only *Haemophilus* colonies on CA and CA-PT were big enough to be measured, showing no significant differences between them (Figure 1B).

### 2.2. Comparison of Microbiological and Physical Properties of Chocolate Agar Prepared with Ground and Irradiated BP vs. Conventional Chocolate Agar

**Figure 2:**
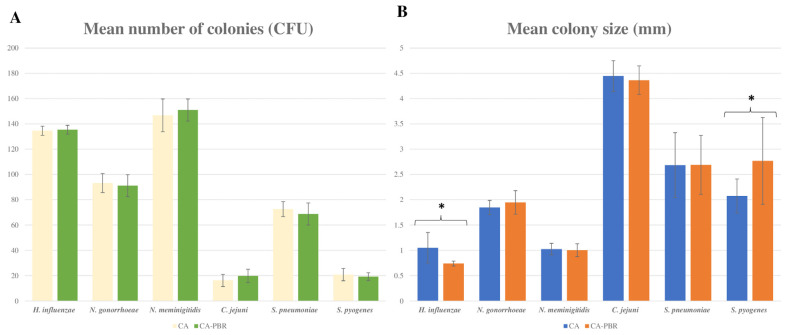
Comparison of CA and CA-PBR media. (**A**) Bar graph showing the colony count (mean CFU). (**B**) Bar graph showing the colony size (mm). * *p* < 0.05. Strains used are: *Haemophilus influenzae* (ATCC 10211), *Neisseria meningitidis* (ATCC 13090), *Neisseria gonorrhoeae* (ATCC 19424), *Campylobacter jejuni* (ATCC 33291), *S. pneumoniae* (ATCC 49619), and *S. pyogenes* (ATCC 19615).

**Figure 3:**
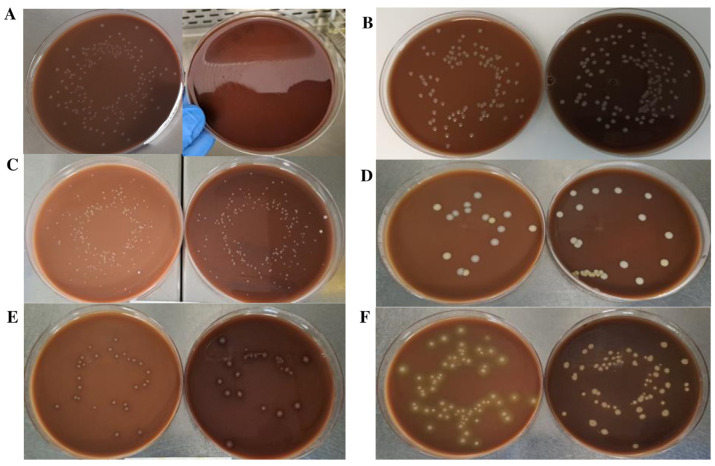
Comparison of CA and CA-PBR productivity properties. Different bacteria growing on CA (left) and CA-PBR (right) media. (**A**) *Haemophilus influenzae* (ATCC 10211). (**B**) *Neisseria gonohrroeae* (ATCC 19424). (**C**) *Neisseria meningitidis* (ATCC 13090). (**D**) *Campylobacter jejuni* (ATCC 33291). (**E**) *Streptococcus pyogenes* (ATCC 19615). (**F**) *Streptococcus pneumonia* (ATCC 49619).

**Figure 4:**
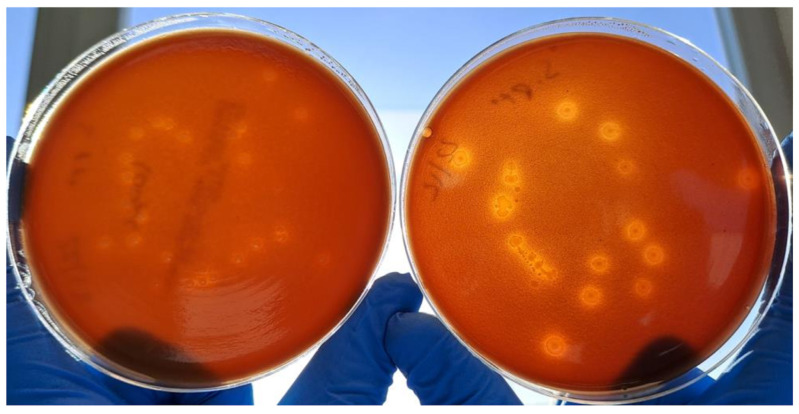
Comparison of *Streptococcus pyogenes* (ATCC 19615) growth on CA (**left**) and CA-PBR (**right**) media.

Conventional CA and CA prepared with BP subjected to a grinding and gamma irradiation process (CA-BPR) were compared to assess the productivity properties of the new medium (Figure 2). Unlike the medium produced with autoclaved BP, CA-BPR had a similar appearance to CA, with a browner background (Figure 3 and Figure 4). Regarding microbial growth, no statistically significant differences were observed in the CFU numbers between CA and CA-BPR media for bacteria with atmospheric requirements for growth: *N. gonorrhoeae* (*p* = 0.705), *N. meningitidis* (*p* = 0.566), *H. influenzae* (*p* = 0.730), *Campylobacter jejuni* (*p* = 0.289), *Streptococcus pneumoniae* (*p* = 0.445), and *Streptococcus pyogenes* (*p* = 0.552) (Figure 2 and Figure 3). The colony size was higher for *H. influenzae* in the conventional medium (1.05 ± 0.30 vs. 0.73 ± 0.04; *p* = 0.010; Student’s t-test for independent data) (Figure 2B and Figure 3A), and higher for *S. pyogenes* in the CA-BPR medium (2.07 ± 0.33 vs. 2.76 ± 0.85; *p* = 0.034; Student’s t-test for independent data) (Figure 2B and Figure 3E). The morphology of the different bacterial colonies did not differ significantly between CA and CA-BPR (Figure 3).

Additionally, signs of β-hemolysis with the *S. pyogenes* strain were more visible in CA-BPR than in CA (Figure 4). On the other hand, CA media seeded with *S. pneumoniae* showed higher α-hemolysis than CA-BPR (Figure 3F).

**Figure 5:**
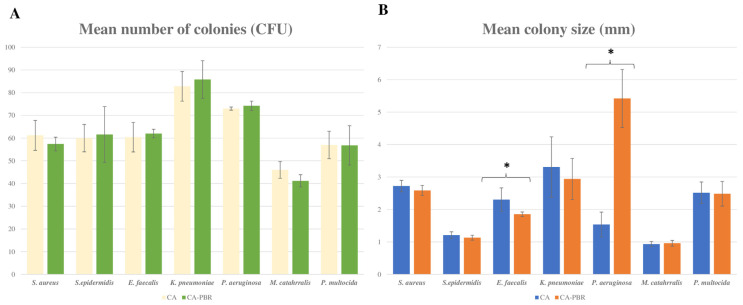
Comparison of aerobic strains grown on CA and CA-PBR media. (**A**) Bar graph showing the colony count (CFU). (**B**) Bar graph showing the colony size (mm). * *p* < 0.05. Strains used are: *Staphylococcus aureus* (ATCC 25923), *Staphylococcus epidermidis* (ATCC 12288), *Enterococcus faecalis* (ATCC 19433), *Klebsiella pneumoniae* (ATCC 13833), *Pseudomonas aeruginosa* (ATCC 9027), *Moraxella catarrhalis* (ATCC 25238), and *Pasteurella multocida* (ATCC 43137).

**Figure 6:**
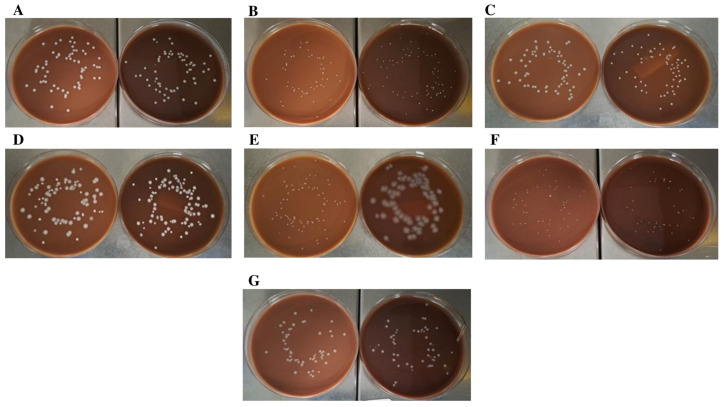
Productivity properties of CA-PBR medium. Aerobic microorganisms growing on CA (left) and CA-PBR (right) media. (**A**) *Staphylococcus aureus* (ATCC 25923). (**B**) *Staphylococcus epidermidis* (ATCC 12288). (**C**) *Enterococcus faecalis* (ATCC 19433). (**D**) *Klebsiella pneumonia* (ATCC 13833). (**E**) *Pseudomonas aeruginosa* (ATCC 9027). (**F**) *Moraxella catarrhalis* (ATCC 25238). (**G**) *Pasteurella multocida* (ATCC 43137).

In an additional study to assess the growth of other species in CA-BPR, equivalence between CA and CA-BPR was observed in terms of CFU counts for *Pasteurella multocida* (57 ± 6.04 vs. 56.80 ± 8.64; *p* = 0.967), *Moraxella catarrhalis* (46.00 ± 3.67 vs. 41.2 ± 2.68; *p* = 0.0504), *Staphylococcus aureus* (61.2 ± 6.53 vs. 57.4 ± 3.04; *p* = 0.283), *Staphylococcus epidermidis* (60.00 ± 6.04 vs. 61.60 ± 12.25; *p* = 0.802), *Enterococcus faecalis* (60.4 ± 6.50 vs. 62.00 ± 1.87; *p* = 0.619), *Klebsiella pneumoniae* (82.8 ± 6.53 vs. 85.80 ± 8.28; *p* = 0.542), and *Pseudomonas aeruginosa* (73 ± 0.70 vs. 74.2 ± 2.04; *p* = 0.270) (Figure 5A). The morphology and characteristics of all bacterial colonies were very similar in both media (Figure 6), with two exceptions: *P. aeruginosa*, which had a significantly higher number of colonies and a mucoid morphology in CA-PBR (1.53 ± 0.38 vs. 5.42 ± 0.89; *p* < 0.01) (Figure 5B and Figure 6E) and *E. faecalis*, whose colonies were significantly bigger in CA (2.30 ± 0.36 vs. 1.85 ± 0.07; *p* = 0.003) (Figure 5B and Figure 6C).

## 3. Discussion

CA is an enriched medium that requires defibrinated blood for the growth and isolation of fastidious pathogenic bacteria. Unlike other blood-based media (e.g., Columbia blood agar, Mueller–Hinton agar, etc.), CA does not contain intact RBCs [16,17]. For CA manufacture, RBCs from blood are lysed by heating to release intracellular components such as hemoglobin (Hb), hemin (X factor), and the coenzyme NAD, which are indispensable for the growth of the target bacteria [16].

Although high quality fresh blood has been traditionally considered the best option to prepare CA, its use is associated with several drawbacks, such as high perishability and ease of contamination [14]. Previous studies have shown that the performance of CA in which defibrinated blood is substituted by autoclaved-sterilized BP is comparable to that of conventional CA for the recovery of fastidious and non-fastidious microorganisms. Avoiding the use of defibrinated blood facilitates several manufacturing steps (such as blood conservation and proper handling to avoid cross-contamination) and reduces production costs [4].

Nevertheless, despite the advantages of BP as a CA component, its application also has drawbacks. For instance, BP is not sufficiently soluble to allow proper homogenization in water, preventing the solubilization of Hb derivatives [18]. Furthermore, the high temperature pre-treatment of the BP solution, which is essential for its sterility, frequently results in the formation of precipitates, probably composed of Heinz bodies, as an effect of Hb denaturation after heating [19,20,21]. These precipitates are unable to dissolve and negatively affect the physical properties of the media. Moreover, the use of autoclaved BP can produce contamination throughout the operation circuit if not handled carefully.

Therefore, the aim of this study was to find an alternative BP pre-treatment process for the manufacture of a medium with equivalent or improved physical and microbiological properties to those of conventional CA. The BP used was blood meal, a product collected during the slaughter of various livestock species (cows in this case) and subjected to a drying process. Blood meal is often used in animal feeds and fertilizer due to its high protein content [22,23]. Given that this product is a by-product of blood and the drying method applied is simpler than lyophilization, its cost is much lower than lyophilized Hb powder (8–45 EUR/g for lyophilized Hb versus 0.026 EUR/g for blood meal) (Merck, https://www.sigmaaldrich.com/ES/es/product/sigma/h2500 (accessed on 1 January 2023)). Moreover, the cost of one plate prepared with ground and irradiated BP would be significantly cheaper (a thousandfold) than that of one plate prepared with defibrinated blood, which represents a substantial saving for laboratories using a high volume of this medium.

It can be assumed that both blood components (blood meal and lyophilized Hb) contain the intracellular components of RBCs necessary for the growth of fastidious bacteria, which are released when RBCs are lysed during the dehydration process performed by the manufacturer and during autoclaving before addition to the medium [24]. In this context, we hypothesized that gamma irradiation, a commonly used sterilization method for pharmaceutical and food products [25,26,27], would degrade Hb protein, stimulating the release of hemin [28,29] without the formation of precipitates, which negatively affect CA medium production. However, the poor growth obtained suggests the medium prepared with irradiated BP (CA-PR) provided a much lower hemin concentration than the one containing autoclaved BP (CA-PT). A possible explanation is that Hb protein denaturation requires a high-temperature environment for hemin release, according to the Teichmann test [30], and thus, hemin formation would occur to a lesser extent in the CA-PR medium. It can be conjectured that irradiation can produce conformational changes in Hb, but not enough Hb degradation to release hemin.

At this point, it was decided to apply an additional pre-treatment to the BP solution before the irradiation process: cell rupture using a blender at 700 W for 5 min. It was envisaged that the resulting RBC breakage would allow different growth factors to be released in a comparable way to the application of high temperatures. This led to the interesting finding that, unlike gamma irradiation alone, grinding plus irradiation produces a medium with equivalent properties to CA, probably due to increased RBC rupture.

To test the properties of this new medium (CA-BPR), the growth of several fastidious and non-fastidious microorganisms was evaluated: *Haemophilus* spp. and *Neisseria* spp., which have specific growth requirements [16,31,32], and *Streptococcus* spp., *Moraxella* spp., and *Pasteurella* spp., whose growth requirements are less strict and can also grow on blood agar [31,32,33]. Another fastidious bacterium known for its complex growth requirements is *C. jejuni* [34,35]. Among non-fastidious bacteria, a representation of species known for exhibiting antimicrobial resistance and causing hospital-acquired infections were evaluated [36,37], including *P. aeruginosa*, *S. aureus* [38,39], *K. pneumoniae*, and *E. faecalis* [40,41,42].

For all the evaluated bacteria, fastidious and non-fastidious, similar growth was obtained on both CA and CA-BPR media. Differences in colony size were observed between some strains: *Haemophilus* colonies were significantly smaller in CA-BPR, while *S. pyogenes* colonies were larger. A possible explanation for these size differences is that the grinding and irradiation processes used to prepare CA-BPR induced less RBC breakage than the high temperature applied in CA. This difference would also explain the higher β-hemolysis of *S. pyogenes* in CA-BPR (Figure 4). Further studies should be performed to validate the modified medium (CA-BPR) with real clinical samples.

It should be noted that when preparing CA-BPR, foam can form in the container during the grinding of the BP solution, which can hinder the collection of the liquid and cause a fraction of solution to be lost. This process should be improved in further validation studies of CA-BPR manufacture.

We believe that the protocol to prepare CA-BPR will be reproducible, and thus, suitable for implementation in different laboratories. As blood meal is a commercial product (cow blood subjected to a drying process), its quality should be homogeneous among different batches, with the guarantee of the manufacturer. The irradiation of BP can be performed using the same conditions in a reproducible manner by a company providing irradiation services.

In summary, the substitution of autoclaving by grinding and gamma irradiation in the pre-treatment processing of BP as a CA supplement resulted in equivalent bacterial growth to that of the conventional medium without the inconvenient low solubility otherwise associated with BP. This more practical and economical methodology could potentially facilitate large-scale CA manufacture or be used in developing countries where fresh defibrinated blood is inaccessible. Further research is needed to optimize and validate the process for industrial application.

## 4. Material and Methods

### 4.1. Media Preparation

Four different chocolate agar media were prepared with: (1) bovine defibrinated blood as a control (CA); (2) bovine BP sterilized by autoclaving at 121 °C for 15 min (CA-PT); (3) bovine BP sterilized with gamma irradiation (CA-PR), and (4) bovine BP subjected to a grinding process using a blender for 5 min and sterilized with gamma irradiation (CA-PBR). The BP was composed of 80% protein and 2.7 g/kg of iron (European Pet Pharmacy, Sweden). Except for the blood supplement, all media were prepared with identical ingredients and conditions, as listed in Table 1.

CA medium was prepared on a large scale using bovine defibrinated blood and gonococcus agar base (Reactivos para Diagnóstico S.L., Sentmenat, Spain). The gonococcus base was dissolved in distilled water and autoclaved at 121 °C for 15 min in a reactor with constant agitation. After autoclaving, the media was cooled to 87 °C and the defibrinated blood of ovine origin was aseptically added. As a final step, the medium was further cooled to 44 °C and a growth promotion supplement (NAD, L-cystine, cysteine, adenine, vitamin B1, vitamin B12, L-glutamine, guanine, p-aminobenzoic acid, carboxylase, ferric nitrate, and glucose) was added to the mix to enhance the growth of fastidious bacteria.

The CA-PT medium was prepared on a small scale under the same conditions as the CA medium, except that ovine defibrinated blood was substituted by autoclaved BP (at 121 °C for 15 min) previously dissolved in water. When the medium was cooled to 50–60 °C, the autoclaved BP was added aseptically with constant agitation.

The CA-PR medium was prepared on a small scale under the same conditions as the CA-PT medium, except the BP was sterilized using ionizing irradiation (gamma irradiation of Cobalt-60, 8–14 kGy dose).

The CA-PBR medium was prepared on a small scale under the same conditions as the CA-PR medium, with the difference that the BP solution was also ground in a blender (700 W for 5 min) before being irradiated. For the irradiation, 5 g of BP were dissolved in 50 mL of distilled water and the resulting suspension was ground and irradiated at 8–14 kGy (Aragogamma, Barcelona, Spain). The irradiated solution (50 mL) was added together with the growth promotion supplement to the autoclaved mix containing the other of ingredients.

For all culture media, 20 mL volumes of the final mixture were dispensed into Petri dishes of 90 mm diameter in a laminar flow room. Plates were packed in airtight containers and stored at 4 °C until use.

### 4.2. Inocula Preparation and Seeding

Inocula were prepared according to ISO 11133:2014/amended 1:2018 [43]. The tested bacterial strains were obtained directly from a reference culture collection (American Type Culture Collection, ATCC, Rockville, MD, USA). A single subculture from the reference strains was used to obtain reference stock strains from which stock and working cultures were prepared using non-selective media: Trypticase soy agar slant tubes for *E. faecalis*, *S. aureus*, *S. epidermidis*, *P. aeruginosa*, and *K. pneumoniae*; chocolate agar for *Campylobacter* and *Neisseria* strains; and blood agar for *Streptococcus* strains, *Moraxella catarrhalis*, and *Pasteurella multocida*. Each strain was verified before use in the appropriate selective and differential media according to ISO 17025:2017 [44].

Stock dilutions were prepared and adjusted using turbidimetry (McFarland unit of 0.5 for productivity assays) (Densitometer DEN-1B, Grant instruments, Cambridgeshire, UK), from which serial dilutions were prepared in the same diluent. For the productivity assays, serial dilutions were prepared to obtain 20–200 CFU per plate.

The strains used to compare growth on CA, CA-PT, and CA-PR media were *Neisseria meningitidis* ATCC 13090, *Neisseria gonorrhoeae* ATCC 19424, and *Haemophilus influenzae* ATCC 10211, as recommended by various guidelines [4,45,46]. To test the productivity properties of the modified media, chocolate agar with defibrinated blood (CA) was used as the standard medium (Table 1).

The strains used to compare growth on CA and CA-PBR media are shown in Table 2, including species with atmospheric growth requirements (*Neisseria meningitidis* ATCC 13090, *Neisseria gonorrhoeae* ATCC 19424, *Haemophilus influenzae* ATCC 10211, *Campylobacter jejuni* ATCC 29428, *Streptococcus pneumonia* ATCC 49619, and *Streptococcus pyogenes* ATCC 19615) and strains without specific growth requirements (*Staphylococcus aureus* ATCC 25923, *Staphylococcus epidermidis* ATCC 12228, *Enterococcus faecalis* ATCC 19433, *Klebsiella pneumoniae* ATCC 13883, *Pseudomonas aeruginosa* ATCC 9027, *Pasteurella multocida* ATCC 43137, and *Moraxella catarrhalis* ATCC 25238).

Volumes of 50–100 μL were plated onto chocolate media using the spiral pour method (Eddyjet, IULmicro; Barcelona, Spain). Plates were incubated at 36 ± 2 °C for periods of 24 h (except for *C. jejuni*, which was incubated at 41.5 °C for 48 h) at different atmospheric conditions, depending on the strain (Table 2). In all cases, recently prepared plates without inoculum were incubated for 2 days without any signs of contamination. For the statistical comparison, 5 replicates of each culture were performed.

### 4.3. Colony Count

The colony count was performed using an automatic colony counter (SphereFlash^®^, IULmicro, Barcelona, Spain). The sizes of colonies were obtained first in pixels and then converted to mm using the automatic colony counter and the mobile application Pixel Measure 1.0 (Leroy Hopson Apps, Vietnam).

### 4.4. Statistical Analysis

Each strain was plated 5 times on the same day. The numbers of CFU obtained in the different media were compared using the Student’s t-test for independent data.

For the statistical comparison of the colony sizes (mm), 10 random colonies were selected from one plate of each sample and compared using the Student’s t-test for independent data. All data were analyzed using a general linear model with SPSS v.21.0 (IBM Corp., Chicago, IL, USA).

In all tests, the significance level was set as 0.05.

## Figures and Tables

**Table 1:** List of ingredients used to prepare the different media. Compounds marked with * were added to the mix after autoclaving the other ingredients.

Ingredient	Chocolate Agar (CA) g/L	Chocolate Agar with BP (CA-PR/CA-PT/CA-BPR) g/L
Special peptone	15	15
Yeast extract	10	10
Starch	1	1
Dextrose	1.5	1.5
Sodium chloride	5	5
Sodium bicarbonate	0.15	0.15
Potassium phosphate	1	1
Dipotassium phosphate	4	4
Agar	12	12
Growth Promotion Supplement * (NAD, L-cystine, cysteine, adenine, vitamin B1, vitamin B12, L-glutamine, guanine, p-aminobenzoic acid, cocarboxilase, ferric nitrate and glucose)	2.75	2.75
Blood powder *	0	10
Defibrinated ovine blood *	50 mL	0

All ingredients were provided by RPD S.L., except for blood powder (European Pet Pharmacy, Sweden) and defibrinated ovine blood (TCS Biosciences Ltd., Buckingham, UK).

**Table 2:** List of strains used to test productivity of CA-PT and CA-PBR media.

List of Microorganisms	ATCC ID	Atmospheric Conditions
*Neisseria meningitidis*	13090	3.5–9% CO_2_
*Neisseria gonorrhoeae*	19424	3.5–9% CO_2_
*Haemophilus influenzae*	10211	3.5–9% CO_2_
*Campylobacter jejuni*	29428	2.5–9.5 % CO_2_
*Streptococcus pyogenes*	19615	3.5–9% CO_2_
*Streptococcus pneumoniae*	49619	3.5–9% CO_2_
*Staphylococcus aureus*	25923	Aerobiosis
*Staphylococcus epidermidis*	12228	Aerobiosis
*Enterococcus faecalis*	19433	Aerobiosis
*Klebsiella pneumoniae*	13883	Aerobiosis
*Pseudomonas aeruginosa*	9027	Aerobiosis
*Pasteurella multocida*	43137	Aerobiosis
*Moraxella catarrhalis*	25238	Aerobiosis

## Abbreviations

Abbreviation	Complete phrase
BP	Blood powder
NAD	Nicotinamide adenine dinucleotide
Hb	Hemoglobin
CA	Conventional chocolate agar
CA-PR	Chocolate agar with blood powder treated with irradiation
CA-PT	Chocolate agar with blood powder treated with temperature
CA-BPR	Chocolate agar with blood powder treated with grinding and irradiation
GC	Gonococcus
GPS	Growth promotion supplement
ISO	International Organization for Standardization
spp.	Species
ATCC	American Type Culture Collection
CFU	Colony-forming unit
RBC	Red blood cells
